# Daytime napping and increased risk of incident respiratory diseases: symptom, marker, or risk factor?

**DOI:** 10.1016/j.sleep.2016.06.012

**Published:** 2016-07

**Authors:** Yue Leng, Nick W.J. Wainwright, Francesco P. Cappuccio, Paul G. Surtees, Shabina Hayat, Robert Luben, Carol Brayne, Kay-Tee Khaw

**Affiliations:** a Department of Public Health and Primary Care, Strangeways Research Laboratory, University of Cambridge, Cambridge, UK; b Division of Mental Health & Wellbeing, Warwick Medical School, University of Warwick, Coventry, UK; c Department of Public Health and Primary Care, Institute of Public Health, University of Cambridge, Cambridge, UK

**Keywords:** Napping, Sleep, Respiratory, Incidence, Cohort study, BMI, Body mass index, CI, Confidence interval, EPIC, European Prospective Investigation into Cancer and Nutrition, HR, Hazard ratio, OSA, Obstructive sleep apnea

## Abstract

**Background:**

We have identified a strong association between daytime napping and increased mortality risk from respiratory diseases, but little is known about the relationship between daytime napping and respiratory morbidity.

**Methods:**

Data were drawn from the European Prospective Investigation into Cancer and Nutrition-Norfolk cohort. Participants reported napping habits during 1998–2000 and were followed up for respiratory disease hospital admissions until March 2009. Cox proportional hazards regression was used to examine the association between daytime napping and respiratory disease incidence risk.

**Results:**

The study sample included 10,978 men and women with a mean age of 61.9 years, and a total of 946 incident respiratory disease cases were recorded. After adjustment for age, sex, social class, education, marital status, employment status, nightshift work, body mass index, physical activity, smoking, alcohol intake, self-reported general health, hypnotic drug use, habitual sleep duration, and preexisting health conditions, daytime napping was associated with an increase in the overall respiratory disease incidence risk (hazard ratio (HR) = 1.32, 95% confidence interval (CI) 1.15, 1.52 for napping <1 h; HR = 1.54, 95% CI 1.14, 2.09 for napping ≥1 h). This association was more pronounced for lower respiratory diseases, especially for the risk of chronic lower respiratory diseases (HR = 1.52, 95% CI: 1.18, 1.96 for napping <1 h; HR = 1.72, 95% CI: 1.01, 2.92 for napping ≥1 h, overall *p* = 0.003).

**Conclusions:**

Excessive daytime napping might be a useful marker of future respiratory disease incidence risk. Further studies are required to confirm these findings and help understand potential mechanisms.

## Highlights

• Daytime napping was associated with 32–54% increase in respiratory incidence risk.
• The association was more pronounced for chronic lower respiratory diseases.
• The association was independent of comorbidities and a proxy measure of sleep apnea.
• Excessive daytime napping might be an early marker of incident respiratory diseases.
• Further studies are needed to help understand potential mechanisms.

## 1 Introduction

Despite growing interest in the influence of comorbid sleep disorders on the progression of respiratory diseases [1], the association between habitual sleep and the onset of respiratory diseases has rarely been studied. Our group has identified an intriguing association between daytime napping and increased mortality risk, particularly from respiratory diseases [2]. While the underlying mechanism remains unclear, the examination of the association between napping and respiratory disease would generate increased interest in the study of napping habits [3], [4]. Daytime napping has been suggested as a marker of obstructive sleep apnea (OSA) [5], but it could also be a more global indicator of respiratory disease. Understanding the link between napping and respiratory morbidity might help with the early detection and control of respiratory disease. We therefore examined the association between daytime napping and the incidence risk of non-fatal respiratory diseases in the European Prospective Investigation into Cancer and Nutrition (EPIC)-Norfolk prospective cohort study [6].

## 2 Materials and methods

A total of 25,639 participants recruited through general practice registers attended the baseline health check in1993–1997 and were followed up for health outcomes. As part of the follow-up, participants were sent questionnaires for completion and returned by post. The Norwich District Ethics Committee approved the study and all participants gave signed informed consent. During 1998–2000, 16,374 participants completed the following question “Do you normally take a nap during the day?” and were asked to indicate the duration of their nap as either <1 h or ≥1 h if they reported napping. We obtained data on all hospital admissions through linkage with the National Health Services health district database. The UK Office of National Statistics flagged all participants according to the International Classification of Disease 10th Revision (ICD-10). All respiratory diseases were defined as J00-J99 and were subdivided into chronic lower respiratory diseases (J40-J47), lower respiratory infections (J10-J22, J85), and upper respiratory diseases (J00-J06, J30-J39). The current analysis presents hospital admissions for respiratory disease followed up from January 2000 until March 2009.

The association between daytime napping (summarized as no napping, napping <1 h/day, and napping ≥1 h/day) and respiratory disease incidence risk was examined using Cox regression. All covariates were chosen a priori and have been described in detail previously [2]. Analysis was confined to participants without self-reported respiratory diseases at the baseline and those with complete data on all covariates. The fully adjusted model included sociodemographic factors, body mass index (BMI), health-related behaviors, self-reported general health, habitual sleep duration, and comorbidities. The comorbidities included stroke, myocardial infarction, diabetes, cancer, and a proxy measure of OSA, with participants who were in the highest BMI quartile and who reported taking antihypertension drugs being defined as likely to have underlying OSA. Analyses were implemented in Stata, version 12.0 (StataCorp LP, College Station, Texas).

## 3 Results

**Fig. 1:**
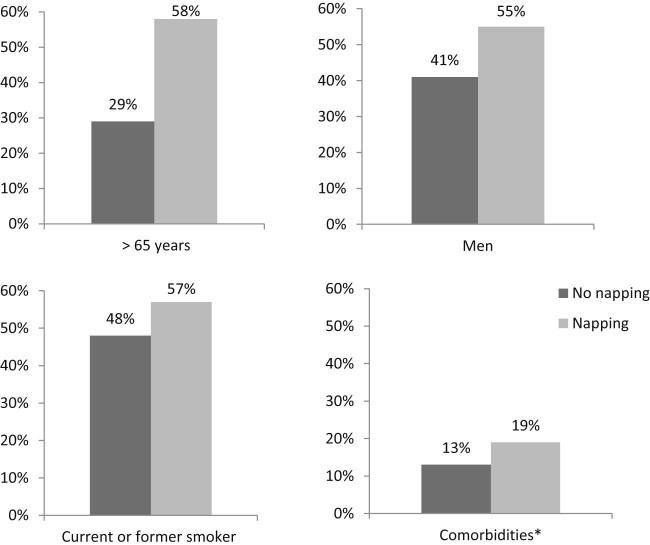
Percentage of baseline characteristics by napping habits in 10,978 men and women, EPIC-Norfolk, United Kingdom, 1998–2000. *Stroke, myocardial infarction, cancer, and underlying sleep apnea. The association between napping and each characteristic was tested by Pearson's χ2 square test, all *P *<* *0.001.

After excluding participants with a history of asthma, bronchitis, and emphysema and those reporting chronic obstructive pulmonary disease (COPD) drug use at the baseline (*n* = 2773), the study sample consisted of 10,978 participants (4903 men and 6075 women, mean age 61.9 ± 9.0 years) with complete data on all covariates. At the baseline, 1700 (35%) men and 1400 (23%) women reported taking naps. Fig. 1 shows the percentage of certain baseline characteristics by napping. Those who reported napping were older, more likely to be men, smokers, and have comorbidities. Supplemental Table S1 summarizes the detailed relationship between covariates and napping habits. Those who reported long napping were more likely to have lower education, higher BMI, poorer general health, longer sleep duration, and be less active.

A total of 946 incident respiratory disease hospital admissions (including 286 from chronic lower respiratory diseases, 452 from lower respiratory infection, and 146 from upper respiratory diseases) were recorded over 9.25 years of follow-up. After adjustment for age and sex, napping was associated with a 40% (for napping <1 h/day) to 94% (for napping ≥1 h/day) increase in the overall respiratory disease incidence risk (Table 1). The association remained even after adjustment for all covariates (hazard ratio (HR) = 1.32, 95% confidence interval (CI) 1.15, 1.52 for napping <1 h; HR = 1.54, 95% CI 1.14, 2.09 for napping ≥1 h). This association was more pronounced for lower respiratory diseases, especially chronic lower respiratory diseases (for napping ≥1 h, HR = 1.72, 95% CI: 1.01, 2.92; overall *p* = 0.003).

When we examined two major chronic lower respiratory diseases separately, daytime napping (of any length) was associated with the risk of both COPD (HR = 1.64, 95% CI 1.17, 2.30) and asthma (HR = 1.50, 95% CI 1.07, 2.09) after multivariable adjustment.

## 4 Discussion

In this large prospective study of middle- to older-aged British adults, napping was associated with a 32–54% increase in the incidence risk of respiratory disease hospital admissions, independent of smoking, comorbidities, and habitual sleep duration. The risk of chronic lower respiratory disease was more than 70% higher among those who napped ≥1 h per day than among those who did not nap.

To our knowledge, this is the first study to report an association between daytime napping and increased incident respiratory diseases. Detailed hospital records on incident respiratory diseases were obtained over a follow-up period of more than 9 years, and subtypes of respiratory diseases were examined to help understand the mechanisms. The effect sizes were reasonably large, even after controlling for the effects of a range of covariates, including smoking and comorbidities. These new results add to our earlier findings on the association between napping and increased respiratory mortality risk [2] and lend further support to the potentially important role of identifying excessive daytime napping for the early detection of respiratory diseases in primary care settings.

Previously discussed study limitations [2] include potential reporting bias and the crude categorization of napping durations, which might not capture the effects of power naps and could have diluted the overall associations. However, we observed a consistently increased overall respiratory risk associated with increasing daytime napping durations. Another limitation concerns the generalizability of these findings, following inclusion of participants with complete data on all covariates. Included participants were younger and had a more favorable socioeconomic and health profile than the rest of the population. While this might have influenced the external validity of the study, within-population associations were unlikely to have been biased as the incidence of respiratory diseases was similar between those included and excluded from the analysis. Finally, sleep-related breathing problems were not recorded in this study. We have used a proxy measure, combining two strong correlates of OSA [7], [8], [9], to account for the effect, but the possibility of residual confounding cannot be ruled out. However, OSA is underdiagnosed and poorly characterized in the general population [10], whereas excessive daytime napping is a noticeable behavior. Therefore, even if some of the observed associations might have been attributed to OSA, excessive daytime napping is potentially useful as a clinical marker in predicting future respiratory hospital admission risk.

Although we are aware of no other studies with which our findings could be directly compared, the present study complements our previous finding [2] that highlighted an increased respiratory mortality risk associated with napping. The Nurses' Health Study II suggested an increased pneumonia risk among those with extreme sleep duration [11], possibly through the influence on immune function. We also found the association between napping and respiratory disease more pronounced for lower respiratory conditions, although the effect was stronger for chronic lower respiratory diseases rather than respiratory infections. While it is unclear whether a physiological link exists between sleep and the lower respiratory system, the identification of potential mechanisms is crucial. Notably, our findings on napping durations and subtypes of respiratory diseases should be interpreted cautiously due to the relatively small number of cases in certain subcategories, and these intriguing results need to be confirmed by larger prospective studies in the future.

The exact biological effects of daytime napping have not been well studied. Daytime napping has been associated with elevated levels of inflammatory biomarkers [12]; airway inflammation is the major mechanism for chronic lower respiratory diseases [13], [14]. Notably, the purpose of napping was not recorded in the present study, and those who took naps could be suffering from daytime sleepiness due to disturbed nighttime sleep, which could also trigger the inflammatory process [15], [16]. Given the high prevalence of excessive daytime sleepiness reported by COPD and asthma patients [17], it is questionable whether daytime napping could be a symptom of respiratory disorders. However, we excluded participants with a self-reported history of respiratory problems, making it plausible that napping precedes the onset of respiratory diseases. Although the influence of other sleep disorders or unmeasured comorbidities cannot be excluded, daytime napping as a noticeable behavior may be useful as a sensitive marker of future respiratory disease. Further work is required to confirm whether there exists a biological pathway through which napping could lead to incident respiratory diseases.

In conclusion, napping was associated with increased risk of incident respiratory disease, especially the risk of incident chronic lower respiratory diseases. Excessive daytime napping might be a useful marker of increased respiratory risk, and identification of this behavior is potentially important for the early detection of respiratory diseases in clinical practice.

## Conflict of interest

The authors declare that they have no conflict of interest.

The ICMJE Uniform Disclosure Form for Potential Conflicts of Interest associated with this article can be viewed by clicking on the following link: http://dx.doi.org/10.1016/j.sleep.2016.06.012.

Conflict of interest ICMJE Form for Disclosure of Potential Conflicts of Interest form. Conflict of interest

## Figures and Tables

**Table 1:** The associations (hazard ratios) between daytime napping of different lengths and the risk of incident respiratory diseases in 10,978 men and women, EPIC-Norfolk Study, United Kingdom, 2000–2009.

Respiratory diseases (No. of cases)	Total No.	Model 1^a^		Model 2^b^	
		HR	95%CI	HR	95%CI
No napping	7878	1.00	Referent	1.00	Referent
Overall respiratory diseases (*n* = 946)					
Napping <1 h	2831	1.40	(1.21,1.60)	1.32	(1.15,1.52)
Napping ≥1 h	269	1.94	(1.44,2.62)	1.54	(1.14,2.09)
Overall p^c^		<0.001		<0.001	
Chronic lower respiratory diseases (*n* = 286)					
Napping <1 h	2831	1.67	(1.30,2.16)	1.52	(1.18,1.96)
Napping ≥ 1h	269	2.43	(1.44,4.10)	1.72	(1.01,2.92)
Overall *p*^c^		<0.001		0.003	
Lower respiratory Infections (*n* = 452)					
Napping <1 h	2831	1.40	(1.15,1.71)	1.33	(1.09,1.63)
Napping ≥1 h	269	1.81	(1.18,2.77)	1.47	(0.96,2.27)
Overall p^c^		<0.001		0.01	
Upper respiratory diseases (*n* = 146)					
Napping <1 h	2831	1.02	(0.69,1.52)	0.96	(0.64,1.43)
Napping ≥1 h	269	1.78	(0.77,4.08)	1.44	(0.62,3.37)
Overall *p*^c^		0.45		0.69	

*Abbreviations:* CI, Confidence Interval; HR, Hazard Ratio.

a Adjusted for age and sex.

b Adjusted for age, sex, social class, educational level, marital status, employment status, nightshift work, body mass index, physical activity level, smoking status, alcohol intake, self-reported general health, hypnotic drug use, habitual sleep duration and preexisting health conditions.

c Tested by Likelihood Ratio Test.

## Appendix Supplementary material

Table S1 Baseline characteristics by napping of different lengths in 10,978 men and women, EPIC-Norfolk Study, United Kingdom, 2000–2002. Table S1
