# Supplementary material for: Daytime napping and increased risk of incident respiratory diseases: symptom, marker, or risk factor?
Source: Sleep Med. 2016 Jul;23:12–5. doi: 10.1016/j.sleep.2016.06.012 (PMC5066369; doi:10.1016/j.sleep.2016.06.012)
Supplement: Table S1 — Baseline characteristics by napping of different lengths in 10,978 men and women, EPIC-Norfolk Study, United Kingdom, 2000–2002. [file mmc2.docx]

| Characteristics  Supplemental table 1 Baseline characteristics by napping of different lengths in 10,978 men and women, EPIC-Norfolk Study, United Kingdom, 2000-2002 |  | No napping | Napping<1h | Napping≥1h |
| --- | --- | --- | --- | --- |
|  | Total N | No. (%) | No. (%) | No. (%) |
| **Age** |  |  |  |  |
| <63  *** p<0.001 | 5755 | 4775 (61%) *** | 886 (31%) | 94 (35%) |
| ≥63 | 5223 | 3103 (39%) | 1945 (69%) | 175 (65%) |
| **Sex** |  |  |  |  |
| Men | 4903 | 3203 (41%) *** | 1542 (55%) | 158 (59%) |
| Women | 6075 | 4675 (59%) | 1289 (46%) | 111 (41%) |
| **Social class** |  |  |  |  |
| Non-manual | 6974 | 5018 (64%) | 1785 (63%) | 171 (64%) |
| Manual | 4004 | 2860 (36%) | 1046 (37%) | 98 (36%) |
| **Education** |  |  |  |  |
| Lower | 4789 | 3328 (42%) *** | 1329 (47%) | 132 (49%) |
| Higher | 6189 | 4550 (58%) | 1502 (53%) | 137 (51%) |
| **Working status** |  |  |  |  |
| Yes | 4517 | 3783 (48%) *** | 674 (24%) | 60 (22%) |
| No | 6461 | 4095 (52%) | 2157 (76%) | 209 (78%) |
| **BMI** |  |  |  |  |
| ≤26.2 | 5595 | 4211 (54%) *** | 1262 (45%) | 122 (45%) |
| >26.2 | 5383 | 3667 (47%) | 1569 (55%) | 147 (55%) |
| **Physical activity** |  |  |  |  |
| Inactive to moderately inactive | 6101 | 4255 (54%) *** | 1675 (59%) | 171 (63%) |
| Moderately active to active | 4877 | 3623 (46%) | 1156 (41%) | 98 (37%) |
| **Smoking status** |  |  |  |  |
| Current | 877 | 624 (8%) *** | 211 (8%) | 42 (16%) |
| Former | 4640 | 3126 (40%) | 1386 (49%) | 128 (48%) |
| Never smoked | 5461 | 4128 (52%) | 1234 (44%) | 99 (37%) |
| **Self-reported general health** |  |  |  |  |
| Good to excellent | 9332 | 6827 (87%)*** | 2320 (82%) | 185 (69%) |
| Poor to moderate | 1646 | 1051 (14%) | 511 (18%) | 84 (31%) |
| **Habitual sleep duration (hr)** |  |  |  |  |
| <6 | 2384 | 1654 (21%)*** | 679 (24%) | 51 (19%) |
| 6-8 | 7462 | 5515 (70%) | 1812 (64%) | 135 (50%) |
| >8 | 1132 | 709 (9%) | 340 (12%) | 83 (31%) |
| **Comorbidities** |  |  |  |  |
| Yes | 1610 | 1024 (13%) *** | 531 (19%) | 55 (20%) |
| No | 9368 | 6854(87%) | 2300 (81%) | 214 (80%) |
